# BAP1 hereditary cancer predisposition syndrome: a case report and review of literature

**DOI:** 10.1186/s40364-015-0040-5

**Published:** 2015-07-02

**Authors:** Sonja Klebe, Jack Driml, Masaki Nasu, Sandra Pastorino, Amirmasoud Zangiabadi, Douglas Henderson, Michele Carbone

**Affiliations:** Department of Anatomical Pathology, Flinders Medical Centre and Flinders University, Bedford Park, SA 5042 UK; University of Hawaii Cancer Center, 701 Ilalo Street, Bldg A-4R, Rm 450, Honolulu, HI 96813 USA; Department of Respiratory and Sleep Medicine, Flinders Medical Centre, Bedford Park, SA 5042 UK

**Keywords:** BAP1 hereditary cancer predisposition syndrome, Mesothelioma, Melanoma

## Abstract

A 72-year-old woman was diagnosed with uveal melanoma, peritoneal mesothelioma and a primary biliary tract adenocarcinoma. She had a strong family history of mesothelioma as well as other malignancies including renal cell carcinoma. The recently described BAP1 hereditary cancer predisposition syndrome was suspected, but immunohistochemical labeling was not conclusive. Genetic testing confirmed a novel and unusual germline mutation in the ubiquitin hydrolase domain of the *BAP1* gene (p.Tyr173Cys) and the patient was diagnosed with the BAP1 hereditary cancer predisposition syndrome. This case demonstrates the importance of clinically recognizing this rare syndrome and its manifestations, some which are still being characterized. It also highlights the importance of genetic testing in cases where there is a high clinical suspicion, even when screening tests, such as immunohistochemistry, in this case, are inconclusive. The diagnosis of a germline *BAP1* mutation may have important implications for both the patient and their families with regards to further genetic testing and active surveillance programs. Further research is needed to fully understand the extent and clinical implications of this rare cancer syndrome.

## Background

A number of rare cancers, in particular mesothelioma and uveal melanoma have been shown to occur together, being noted as early as the 1970s. Furthermore there have been cases of strong familial clustering of malignant mesothelioma, often with limited asbestos exposure, a known carcinogen recognized as causing mesothelioma. Genetic studies of these families subsequently lead to the discovery of germline mutations in *BAP1* and the recognition of a new cancer predisposition syndrome. At present this syndrome has been shown to predispose to the development of uveal melanoma, malignant mesothelioma, cutaneous melanoma and renal cell carcinoma, but has also been implicated in the development of other malignancies. Here we report the first case of this new cancer syndrome in Australia, leading to the development of 3 distinct malignancies.

## Case presentation

**Fig. 1:**
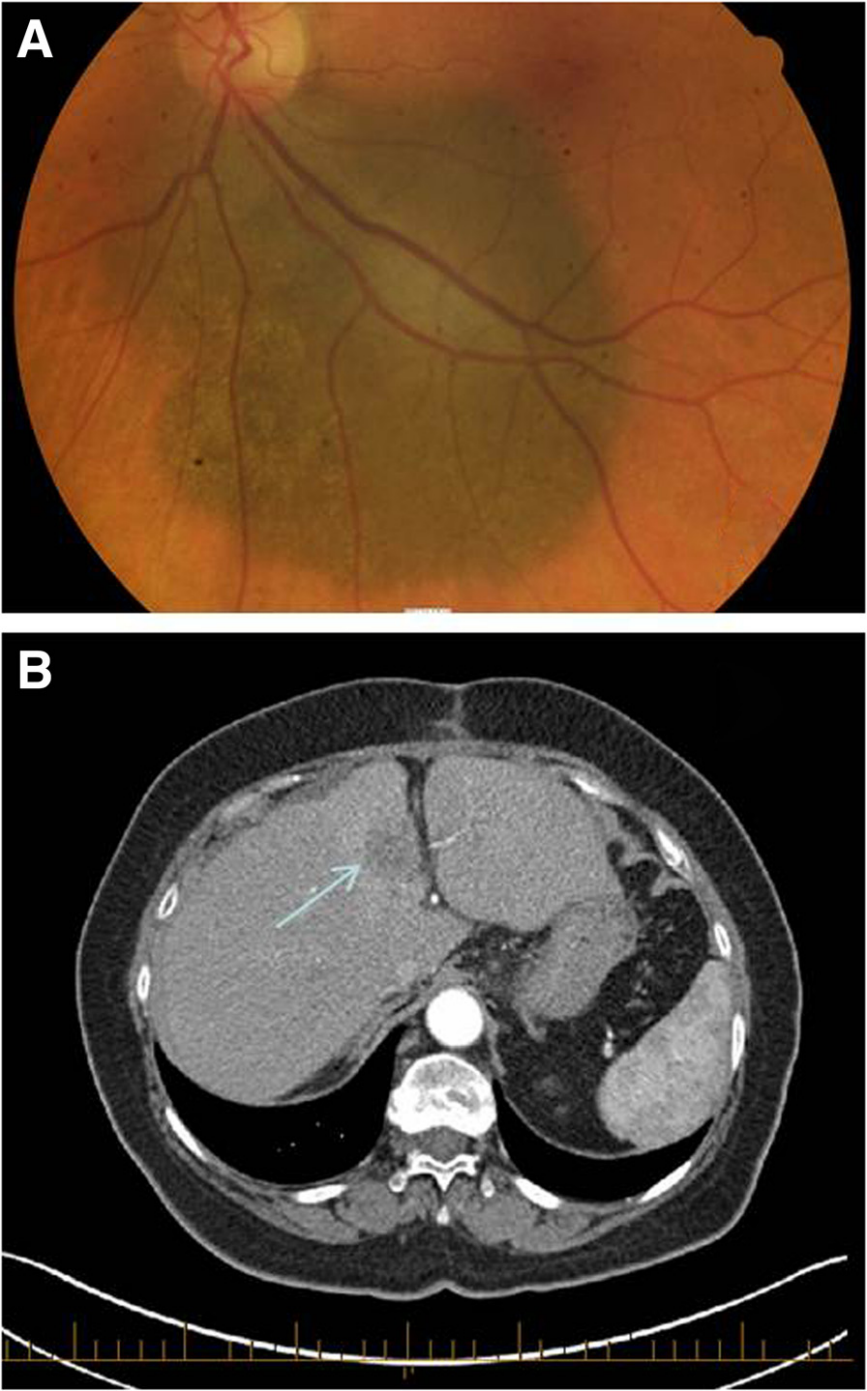
Imaging of patient with germline BAP1 mutation. **a**. Retinal examination revealed a melanocytic tumor with irregular margins involving the majority of the retina. **b**. CT of the abdomen prior to surgery identified a 32 mm lobulated lesion in segment 4B of the liver as indicated by the arrow

A 72-year-old, previously well, woman was found to have left ocular uveal melanoma on ophthalmology review (Fig. 1). She had previously been noted to have ‘freckles’ on her left retina. At the time of diagnosis the patient was asymptomatic, suffered no visual impairment and declined further investigation, radiotherapy or enucleation. A one-year follow up showed growth of the uveal melanoma with staging undertaken prior to intended radiotherapy. An abdominal CT scan demonstrated a lesion within segments 4 and 7 of her liver (Fig. 1). A Further PET scan showed a focal area of FDG avidity within segment 4 but no increased FDG uptake in liver segment 7,

A diagnostic laparoscopy was performed for the liver lesions of unknown origin. Intraoperatively, no free intraperitoneal fluid was seen but numerous peritoneal nodules were noted on the diaphragmatic and peritoneal surfaces, particularly in the right upper quadrant. There was no obvious omental disease. These peritoneal nodules along with the liver lesion were biopsied. The liver biopsy showed focally necrotic poorly differentiated adenocarcinoma. Immunohistochemical labeling was in keeping with cholangiocarcinoma and in the absence of a primary site in the extrahepatic biliary system or upper gastrointestinal tract the lesion was considered to likely represent a primary intrahepatic cholangiocarcinoma. The lesion did not label for any immunohistochemical markers for melanoma. The peritoneal biopsy showed nodular proliferations of mesothelial cells with infiltration into the submesothelial adipose tissue indicative of malignant mesothelioma of epithelial type. Immunohistochemistry was positive for mesothelial markers and negative for carcinoma markers.

**Fig. 2:**
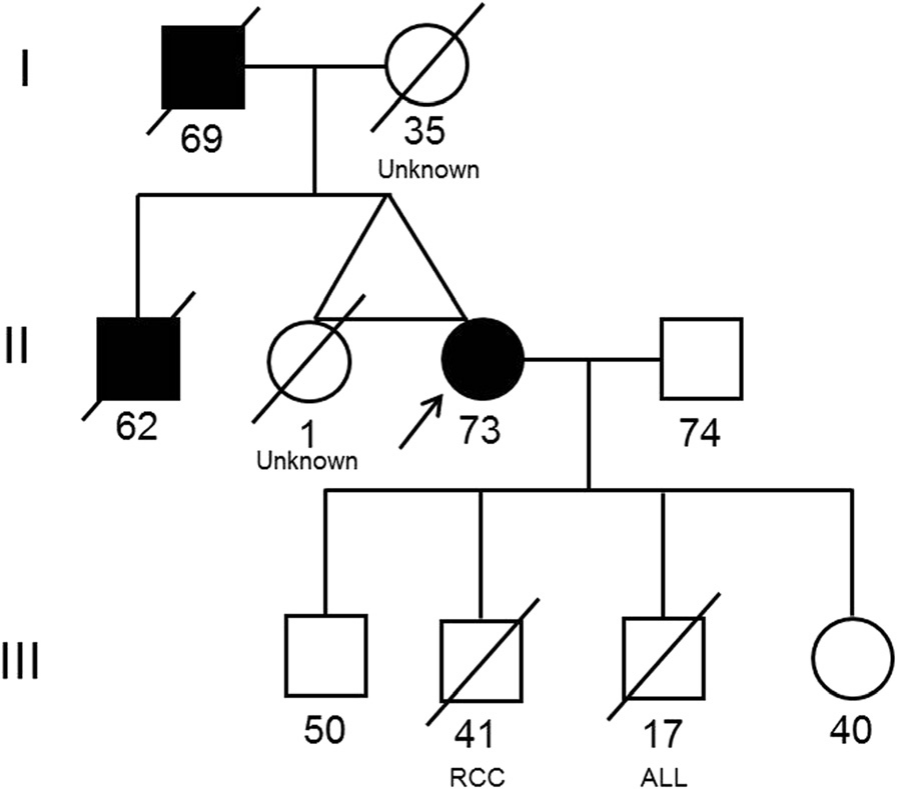
Pedigree of the patient’s family with a BAP1 germline mutation. Black Squares indicate a diagnosis of malignant mesothelioma. Arrow indicates the presence of BAP1 mutation as determined by sequencing studies. Two of the patient’s children passed away with renal cell carcinoma (RCC) and acute lymphocytic leukaemia (ALL)

The patient’s father and brother had both died of malignant mesothelioma (Fig. 2) having had occupational exposure to asbestos. The patient herself had no occupational exposure to asbestos but may have had ongoing household exposure since the 1970s. Her past medical history was unremarkable besides beta thalassemia minor and a previous hysterectomy for benign disease. The patient was of Greek descent. She was a non-smoker and only occasionally drank alcohol. The patient had four children, and two of these had passed away with renal cell carcinoma and acute lymphocytic leukaemia at ages 41 and 17 respectively (Fig. 2).

Elective surgical resection was deemed appropriate for the patient’s primary liver mass. A CT prior to resection demonstrated no change in the liver lesion and no evidence of extrahepatic spread. Intraoperatively, a firm porta hepatic node was identified with frozen sectioning confirming metastatic adenocarcinoma. Fine needle aspiration of the segment 7 also showed adenocarcinoma and in view of positive intra- and extrahepatic disease the liver resection was abandoned. A strip of peritoneum was also removed which again demonstrated malignant mesothelioma.

**Fig. 3:**
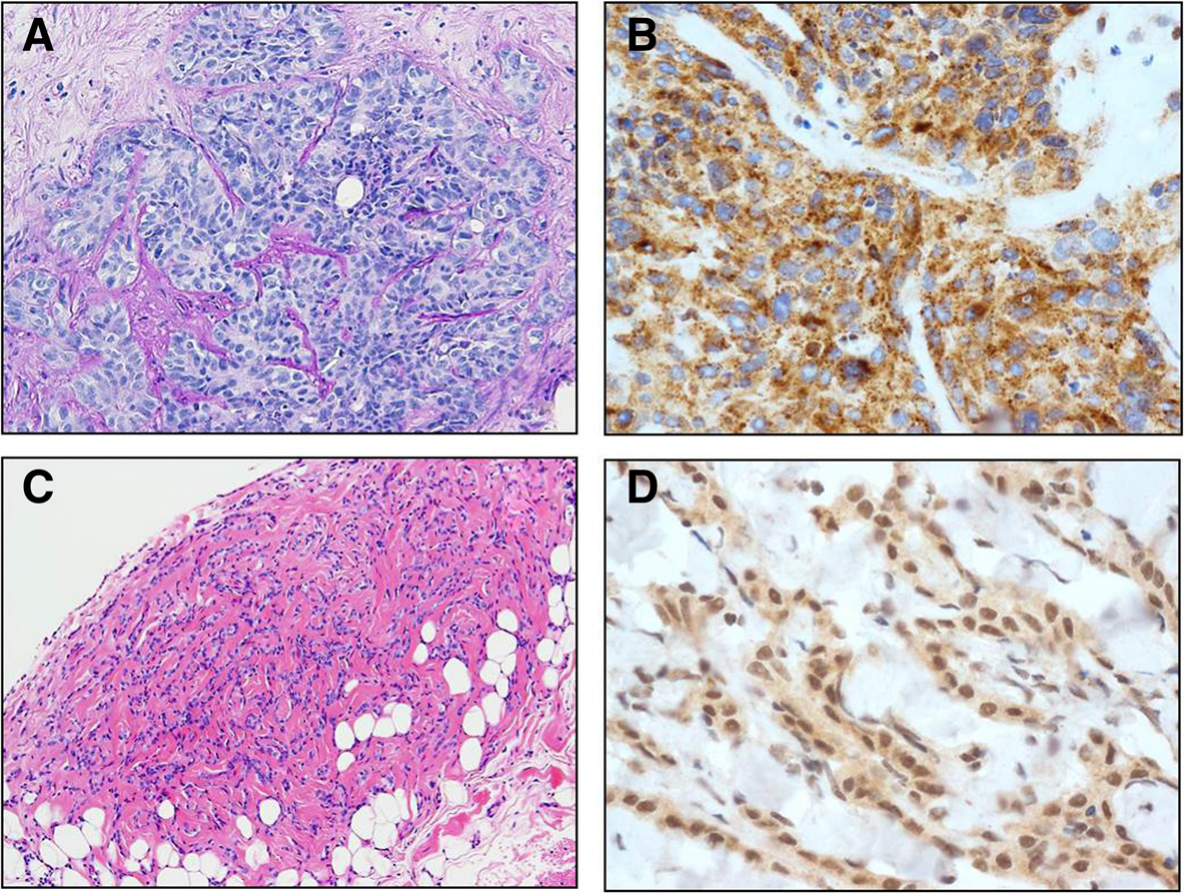
Histological findings of patient with BAP1 germline mutation **a**. Liver biopsy demonstrating focally necrotic poorly differentiated intrahepatic adenocarcinoma (Hemosiderin and Eosin, original magnification 250×). **b**. Immunohistochemical labeling for BAP1 protein in the cholangiocarcinoma, showing loss of nuclear staining. (Santa Cruz BAP1 monoclonal antibody (sc-28383) 1: 200. Dako dual system detection, original magnification 400×) **c**. Peritoneal biopsy demonstrating malignant mesothelioma of epithelial type with invasion into the submesothelial adipose tissue. (Hemosiderin and Eosin, original magnification 250×). **d**. Immunohistochemical labeling for BAP1 for peritoneal biopsy showing retained BAP1 nuclear staining in malignant peritoneal mesothelioma (Tissue preparation s above, original magnification 400×)

**Fig. 4:**
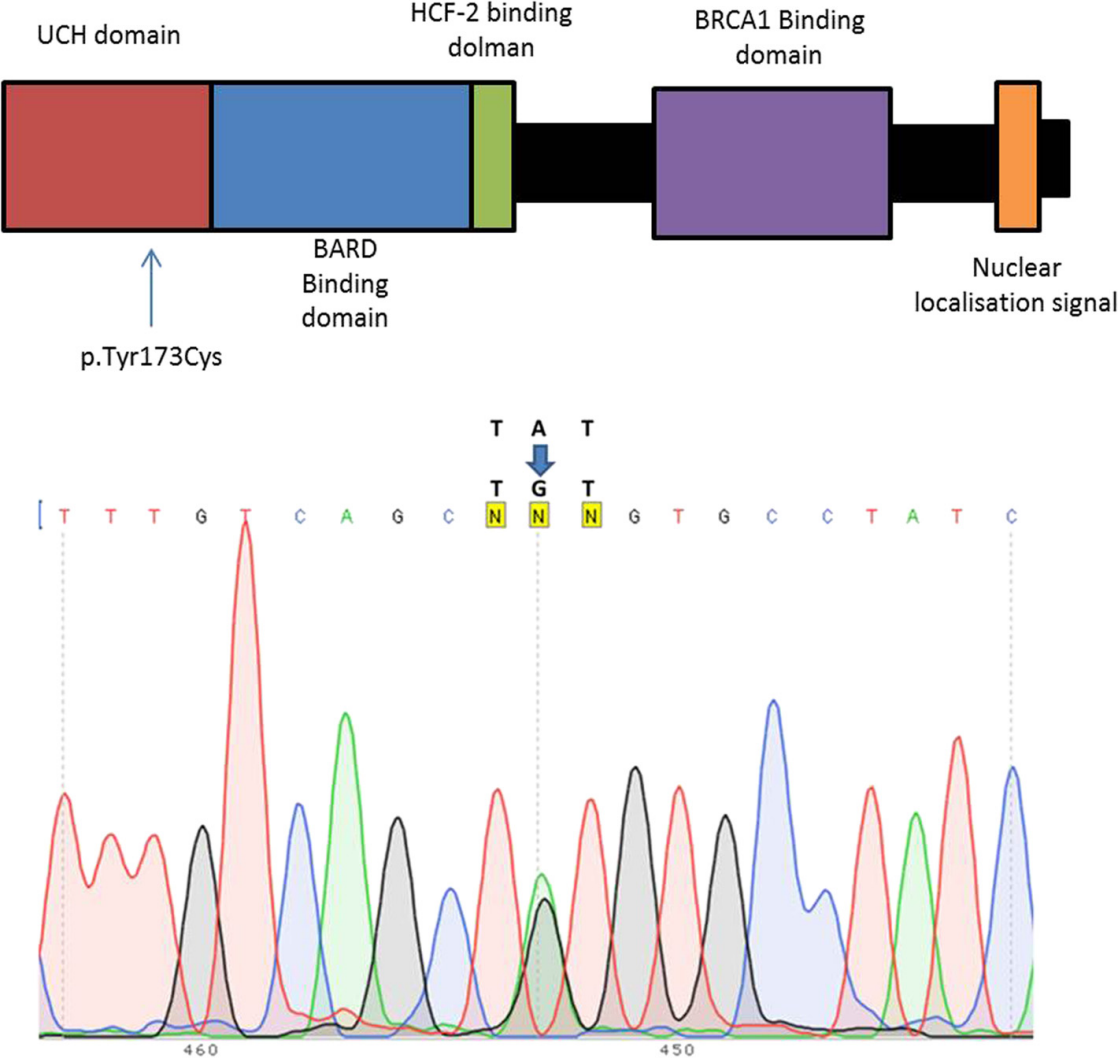
Germline BAP1 missense mutation found in this patient. Germline DNA was extracted from the patient’s blood and analysed by Sanger sequencing. DNA amplification and sequencing were performed as previously described (Forward primer: GGAGTTGGCCAAGGCCCATAATAGC; reverse primer: CCTGGATACTCTCTGTCCCTCCCAAAG) [1]. Electropherogram shows BAP1 missense mutation (g.52441252A > G) within exon 7. This mutation produces an amino-acid sequence switch from tyrosine to cysteine in the ubiquitin hydrolase domain (UCH), and indicated by the diagram

In light of her uveal melanoma and malignant mesothelioma, combined with her family history of mesothelioma it was suspected that the patient might have BAP1 hereditary cancer predisposition syndrome. Testing for *BAP1* mutations by immunohistochemistry showed loss of nuclear BAP1 labeling in the primary biliary tract adenocarcinoma, but nuclear labeling for BAP1 was retained in the malignant mesothelioma (Fig. 3). No biopsies were taken from the uveal melanoma. Germline DNA sequencing was performed and revealed the patient to carry a germline missense mutation in the catalytic domain (g.52441252A > G, p.Tyr173Cys) located in exon 7 of the *BAP1* gene (Fig. 4). This mutation is predicted to generate a non-functional full-length protein, due to impairment of its ubiquitin hydrolase activity.

Further CT surveillance of the patient demonstrated an enlarging liver mass, development of new liver lesions and increasing portacaval lymph nodes. No other distant sites of metastasis where identified on CT. The patient become symptomatic 23 months after the diagnosis of cholangiocarcinoma and mesothelioma and was commenced on palliative chemotherapy. She passed away 31 months after diagnosis of cholangiocarcinoma and mesothelioma due to progression of her intraabdominal malignancies.

## Discussion

BAP1 hereditary cancer predisposition syndrome was first described in 2011 based on investigations of two American families [1]. It is a syndrome that describes a predisposition to the development of several malignancies, in particular uveal melanoma and malignant mesothelioma as well as cutaneous melanomas, basal cell carcinomas and renal cell carcinoma [2-7]. Other cancers have been reported in families suffering germline mutations but no direct correlations have yet been established. In a review of 76 patients with a BAP1 germline mutation, 53 patients had developed at least one malignancy with 13 developing at least two. Those who had not yet developed a malignancy were all aged less than 55 [8]. This study highlights the strong link between germline *BAP1* mutations and carcinogenesis. Here, we reported a case involving an individual with a germline BAP1 mutation who was diagnosed with 3 distinct malignancies in short succession, an event that appears uncommon even for individuals carrying a germline BAP1 mutation.

BAP1 (BRCA1-associated protein 1) is a nuclear protein encoded by the tumour suppressor gene located on chromosome 3p21.1 [9]. It is a deubiquitinating enzyme, usually part of a protein complex, and has been implicated in a number of different cellular processes including chromatin remodeling, cell cycle progression, cell differentiation and DNA damage responses [4]. The understanding of BAP1’s role in cellular processes and subsequent carcinogenesis has increased in recent years however the function of BAP1 is still being fully characterized, in particular its contribution to carcinogenesis. Both germline and somatic mutations have been implicated in tumour progression [1, 3, 9]. *BAP1* mutations may result in proteins that lack the nuclear localization sequence, or may affect its deubiquitinase activity [1, 4, 9]. It is not fully known why germline mutations may predispose to the development of certain cancers over others; most likely cell-type specific functions as well as gene-environment interactions play an important role.

The *BAP1* germline alteration identified in our patient was a missense mutation at the active site of its ubiquitin hydrolase domain. Similar missense mutations were found in uveal melanoma (p.Ser172Arg) [10] and pleural biphasic mesothelioma (p.Tyr173Ser) tissue [11]. While the role of this specific mutation is not known yet, the critical role of the deubiquitinating.activity of BAP1 in its tumour suppressor activities have been well established [12, 8] The tumor suppressor function of BAP1 has been shown to require its nuclear localization. Therefore absence of BAP1 nuclear staining and/or presence of cytoplasmic BAP1 indicate the presence of an abnormal BAP1 protein. Both N-terminus BAP1 mutations and C-terminus mutations lead to lack of BAP1 nuclear labeling due to either lack of the nuclear localization signal, or to lack of deubiquitinase activity, as autodeubiquition is required for nuclear transport of BAP1 protein [13]. This has been confirmed in a comprehensive study showing that all BAP1 abnormalities found in mesothelioma to date are always associated with absence of BAP1 nuclear staining and/or presence of cytoplasmic BAP1 staining [14].

The complete loss of BAP1 nuclear staining observed in our primary biliary tract adenocarcinoma is likely due to LOH at somatic level, while the mesothelioma tissue seems to have preserved the other BAP1 allele. One could argue that the fact that mesothelioma developed even in absence of LOH further confirms the deleteriousness of the Tyr 173 mutation. Definitive sequencing was not performed on tumour samples, which were largely exhausted by diagnostic tests. Loss of nuclear BAP1 staining on immunohistochemistry was thought to be an accessible and reliable test of *BAP1* mutations, including germline [15, 8] and has been used for prognostication in mesothelioma [16, 17]. However the presence of retained nuclear staining in this case highlights the need for clinician awareness of *BAP1* germline mutations and for definitive germline genetic testing.

With regards to specific cancers, germline *BAP1* mutations have been shown to play a role in a small number (3-4 %) of uveal melanomas [18, 19]. Uveal melanoma is a rare ocular cancer with variable outcome, depending on molecular and clinical characteristics [20]. *BAP1* mutations have been implicated in metastatic ability. A germline mutation was found in 4 out of 50 metastatic uveal melanomas and was not identified in a further 50 non-metastatic tumours [18]. Sporadic *BAP1* mutations have also been found in 84 % of metastasizing uveal melanomas [10]. Loss of *BAP1* both germline and sporadic appears to lead to a more aggressive tumour phenotype in uveal melanoma.

Malignant mesothelioma is another rare cancer that has been associated with *BAP1* germline mutations [4]. It is an aggressive cancer with limited treatment options often due to late diagnosis. Both germline and sporadic *BAP1* mutations has been shown to play a role in malignant mesothelioma. *BAP1* appears to be sporadically mutated in a significant portion ~60 % of malignant mesotheliomas [11, 1, 14, 9]. Germline mutations make up much smaller proportion of cancers. A recent study compared the clinical characteristics of 23 mesotheliomas that occurred in carriers of germline BAP1 mutations to 10,556 sporadic mesotheliomas [21]. These patients tended to have a family history of malignant mesothelioma with limited asbestos exposure. Tumors that occur in a setting of germline BAP1 mutations are much less aggressive than their somatic counterparts and are associated with a significant improved mean survival of 5 years compared to 1 year with sporadic malignant mesothelioma [21]. This improved survival is observed in the patient reported here. Loss of nuclear labeling for BAP1 in the absence of known germline mutations has been associated with a less dramatic improvement in prognosis [17, 16]. Germline mutations also had a higher incidence of peritoneal disease (as in our patient) where in sporadic tumours pleural disease predominates. The recent discovery of BAP1 involvement has given much needed insight into the pathogenesis of mesothelioma and in particular has added valuable prognostic information [16, 17].

Germline *BAP1* mutations have cutaneous manifestations that include basal cell carcinomas, malignant melanoma and the recently described melanocytic-BAP1 mutated atypical intradermal tumour (MBAIT) [2, 8, 6, 5, 7]. MBAITs are pink to tan benign skin tumours around 0.2 – 1 cm in diameter that macroscopically resembles dermal nevi but are clinically, histologically, and genetically distinct. They have been shown to have high levels of mutated *BRAF* which can distinguish them from benign Spitz naevi [15]. They can appear as early as the second decade with an individual developing multiple lesions over their lifetime. They appear clinically stable with a low malignant potential but there are reports of progression to malignant melanoma. The number of cutaneous melanoma is still low in these families despite significant number of MBAITs. Bi-allelic loss of *BAP1* in itself along with a *BRAF* mutation is not sufficient for melanoma formation [2]. It has been suggested that MBAITs may be used to screen individuals for germline *BAP1* mutations; 19 out of 35 individuals with a *BAP1* mutation were found to have one or more lesion [4]. Furthermore, MBAITs occur earlier than other associated malignancies, allowing for identification of at risk individuals, genetic testing and implementation of surveillance protocols.

Renal cell carcinoma is the most recent tumour associated with *BAP1* germline mutations. The majority of renal cancers are sporadic however a small proportion (2-4 %) is familial, the majority of these due to Von Hippel Lindau syndrome [22]. A much smaller component can be attributed to germline *BAP1* mutations [3, 23]. It should be noted that our patient’s son died of renal cell carcinoma. Sporadic *BAP1* mutations have also been associated with a more aggressive form of cancer [24] however it appears germline mutations may have different consequences [3]. Further research is needed to understand the contributing and prognostic role of *BAP1* in renal cell carcinoma.

Other cancers including meningioma, lung, ovarian, pancreatic and breast cancers have been found in families that harbor *BAP1* germline mutations [4]. However it is unclear whether germline *BAP1* mutations directly predispose to the development of these tumours.

Our patient was diagnosed with a primary biliary tract cancer. There are reports of individuals with *BAP1* mutations developing primary biliary tract cancers but no definitive predisposition has been attributed [4, 21]. Somatic BAP1 mutations are relatively frequent in cholangiocarcinoma [25-28]. Our own immunohistochemistry showed complete loss of BAP1 nuclear staining in the primary biliary tract adenocarcinoma. This indicates that secondary sporadic mutations had occurred in the adenocarcinoma, resulting in loss of protein translocation (Fig. 3). Further epidemiological studies are needed to confirm whether primary biliary tract tumours, contribute to the spectrum of BAP1 hereditary cancer predisposition syndrome.

The recent discovery of this rare cancer predisposition syndrome has a number of clinical implications. Firstly, it is important to identify these patients and their families through genetic testing. It would be advisable to perform genetic testing on patients diagnosed with uveal melanoma and mesothelioma, those with mesothelioma who have a strong family history of the disease and as well as those found to have MBAITs. The indication of offering BAP1 genetic testing for other familial cancer associations is less clear, considering the rarity of this syndrome. Those who are confirmed to carry a BAP1 mutation will then need appropriate genetic counseling for themselves and their families. Some authors advocate affected family members should undergo annual total skin checks for MBAITs as well as ocular examination [8]. As the full extent of this syndrome is not yet known it is unclear what the appropriate surveillance programs should be. Early detection and management of malignant mesothelioma and other related malignancies may improve survival.

## Conclusion

BAP1 mutations have been described in some US and European families with a very high incidence of cancer and they are transmitted in a Mendelian fashion (i.e., about 50 % of offspring inherit the mutation). So far all of the family members that inherited this mutation have developed one and often several cancers by age 65 (18). Characteristically, when malignancies occur in a setting of germline BAP1 mutations they are less aggressive and patients live significantly longer compared to patients with the same types of tumors occurring in a sporadic setting [21]. Here we describe the first Australian family affected by the BAP1 cancer syndrome. Our findings reveal that this cancer syndrome is ubiquitous and it is not restricted to the US and Europe. Clearly identification of these patients is critical to provide them correct medical advice and genetic counseling.

## Consent

Written informed consent was obtained from the patient for publication of this Case report and any accompanying images. A copy of the written consent is available for review by the Editor-in-Chief of this journal.
